# Chronic Cervicogenic Tinnitus Rapidly Resolved by Intermittent Use of Cervical Collar

**DOI:** 10.3389/fpsyt.2016.00043

**Published:** 2016-03-23

**Authors:** Karl Bechter, Martin Wieland, Gerhard F. Hamann

**Affiliations:** ^1^Clinic for Psychiatry and Psychotherapy II, BKH Günzburg, Ulm University, Günzburg, Germany; ^2^HKM HandelsKontorMeindl GmbH Gundelfingen, Ichenhausen, Germany; ^3^Department of Neurology and Neurological Rehabilitation, BKH Günzburg, Günzburg, Germany

**Keywords:** cervicogenic tinnitus, cervicogenic headache, cervical collar, tinnitus mechanisms, tinnitus treatment

## Abstract

**Introduction:**

Cervicogenic tinnitus is not a generally accepted pathogenetic subtype, which might be subsumed under the concept of somatosensory tinnitus. After the personal experience of therapy-resistant tinnitus in context with a cervical pain syndrome (CPS) and successful add-on treatment with cervical collar (CC), the idea was pursued in several individual treatments in patients.

**Patients and methods:**

Reporting one particular case with chronic tinnitus, considered untreatable, that rapidly improved with exclusive treatment by CC use. Thereafter, tinnitus was experimentally replicated by head inclination, the respective neck–head angles, and cerebral blood flow was measured.

**Results:**

Chronic subjective tinnitus of a 20 years duration completely disappeared within 4 weeks with an intermittent short time application of CC. Thereafter, tinnitus was deliberately again induced by head inclination, set on with anterior tilt of 14°, reaching maximum strength by 23°. Tinnitus stopped with return to neutral head position. Blood flow in the vertebral arteries on both sides was unchanged during head inclination with prevalent tinnitus; however, blood flow was physiologically reduced with head rotation though not accompanied by tinnitus.

**Discussion:**

In a single case of chronic tinnitus, we found that treatment with CC rapidly led to full remission. Blood flow reduction in vertebral arteries was unrelated to tinnitus. However, tinnitus could be resumed by constrained head postures. Experimental tinnitus replication (by inclination) points to an underscored role of upper posterior cervical muscle groups, matching with animal experiments, also in concert with other triggers including psychological factors.

## Introduction

The causes of tinnitus are varied, but remain unclear in most individual cases (1, 2). Cervicogenic tinnitus is presently neither an accepted pathogenetic scenario nor an established subgroup. A search in PubMed and Scopus yielded few positive results for the instructions: “cervicogenic tinnitus,” “tinnitus and muscle tension,” “tinnitus causality,” and “tinnitus etiology.” Cervical mechanisms have been proposed to be involved with different pathomechanisms, most interesting therein appeared to us the model of somatosensory tinnitus (3, 4), supported, at least in part, by randomized study (5).

Years ago, suffering personally (author Karl Bechter) from painful cervical syndrome (CPS) with varying comorbid symptoms mainly tinnitus and vertigo, responding only partially to established treatments (physiotherapy, massage, posture control, exercise), the remaining therapy-resistant symptoms appeared hardly explained, as MR imaging of the cervical spine was normal. These resistant symptoms provided an apparent opportunity of intense self-observation from which a special relationship between CPS and tinnitus/vertigo with varied muscular tension especially of the upper posterior cervical spine was felt to exist. This idea seemed further supported by experimental findings in the literature, which demonstrate a specific role of cervical muscle tension, exclusively the upper posterior muscle groups, to trigger wide spread CNS activity alterations, although tinnitus was not studied herein (see Discussion). Intermittent short time use of cervical collar (CC) appeared effective in the own personal case; therefore, such new treatment was continued as add-on to the established treatments and with this combined treatment a complete remission from all symptoms was achieved in the own case within about 3 months, stable over more than 10 years now. Occasionally, the use of CC was again required (about three times a year), an aspect which rather confirmed the potency of CC use.

Based on these personal observations, we began individual treatments with CC add-on treatment in hospitalized patients of our psychosomatic department, when patients were suffering from CPS (with or without neuroradiological cervical spine abnormalities), which were not rarely accompanied by tinnitus. Based on this hospital case series (in preparation), we report here one particular outpatient case, who suffered exclusively from chronic tinnitus but not actually from CPS, although, the case had begun 20 years ago in association with CPS. The CPS was then successfully treated; however, the tinnitus remained therapy resistant over 20 years until now, despite being treated according to the advice of tinnitus specialists, who soon had concluded the tinnitus was untreatable. In this single case, we, only now, had exclusively applied short time intermittent daily CC treatment, which led rapidly to complete remission of chronic tinnitus. Detailed aspects of this case and especially the experimental replication of tinnitus with controlled head inclination after successful treatment of tinnitus may give some more general hints into tinnitus pathomechanisms potentially with a special role for the upper posterior cervical muscle groups (UPCM) and may suggest new treatment options, the use of CC for tinnitus.

## Patients and Methods

A short series of patients suffering from CPS and comorbid tinnitus were treated in the department psychotherapy and psychosomatics of the Department Psychiatry and Psychotherapy II, Ulm University, in individual case trials in accordance with the Helsinki Declaration with add-on use of CC.

All patients were hospitalized and gave informed consent, after being informed about the prevailing skepticism about CC use in neurotraumatology and unexplored effect in case of tinnitus. The single patient described in the study was similarly fully informed and fully consenting.

**Figure 1:**
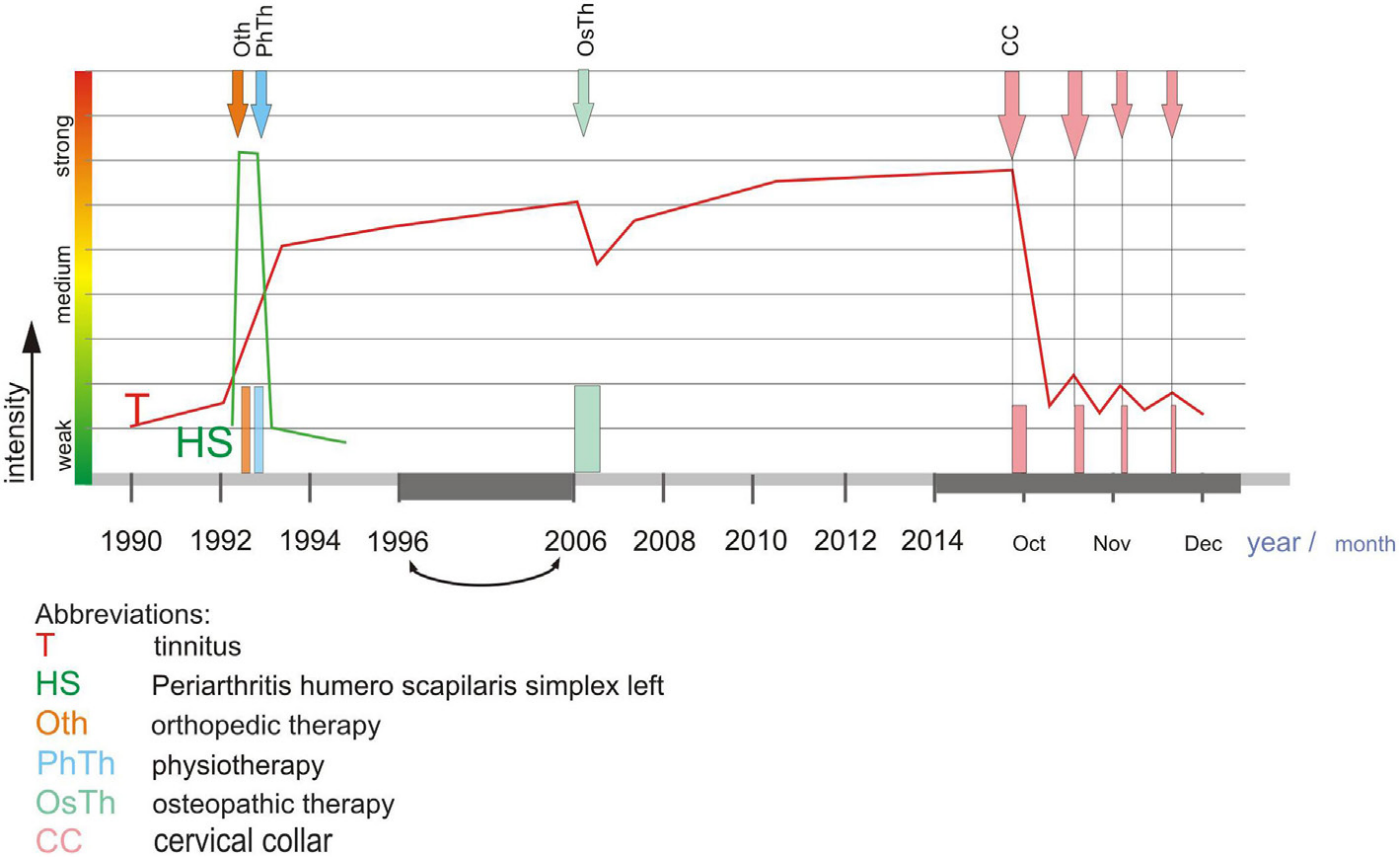
**The figure represents the full length of disease history of this single case, from the beginning 20 years ago with a cervical pain syndrome associated with tinnitus**. When the cervical pain syndrome was intensely treated with established treatments (mainly physiotherapy of various types; for details, see the respective signs within figure) the CPS only gradually improved over time, even worsened after a chiropractic measure. After successful treatment of the cervical pain syndrome, the tinnitus, nevertheless, was chronic, persistent over about 20 years. The tentative recommendation to consider this type of tinnitus as a minor symptom of an incompletely treated cervical syndrome was based on own personal experiences and observations (Karl Bechter, see Introduction), and led to recommend exclusively the intermittent CC use in an outpatient setting (with no other treatment). CC was used repeatedly during day time, initially more frequent, after 2 weeks less frequent, and after 4 weeks the usage nearly ended because of rapid, complete relief from tinnitus. Abbreviations: T, tinnitus; HS, periarthritis humero scapilaris simplex left; Oth, orthopedic therapy; Pth, physiotherapy; OsTh, osteopathic therapy; CC, cervical collar.

Based on this short series of hospitalized patients with prevalent CPS, we report on one particular patient here, although he did not match with the typical symptoms of the case series in many aspects: this patient was not hospitalized and did not suffer from CPS but exclusively from chronic tinnitus, though, in the beginning of his case history, both the tinnitus and CPS were comorbid features. Furthermore, in this particular case, CC treatment was recommended as the exclusive treatment done in the critical time period. Our idea (see Introduction) was that tinnitus may represent, in this case, a type of minor cervical syndrome CPS without pain but with some minor muscle tension prevalent in the craniocervical transition. Interestingly, chronic tinnitus in this patient rapidly and fully remitted to normal within 4 weeks of intermittent CC use. Therefore, the patient himself (coauthor Martin Wieland) wanted to test tinnitus replication under controlled experimental conditions, he had himself observed tinnitus re-onset taking place spontaneously with strong head inclination, and publish the findings in detail for the sake of others. The chronic long term case history, we felt, was informative when including the initial period of comorbid CPS with tinnitus and the treatments performed over the whole time period of tinnitus prevalence of 20 years (see Figure 1).

### Experimental CC Treatment

The CC was individually adapted according to the manufacturer’s recommendation (Basko Healthcare Med Surg Innov Ltd., Lancaster, UK). We recommended the usage of customized CC exclusively for short time periods (15–30 min), that is intermittently over day time, three to five times a day.

### Experimental Tinnitus Replication and Determination of Tinnitus Relevant Head Posture

**Figure 2:**
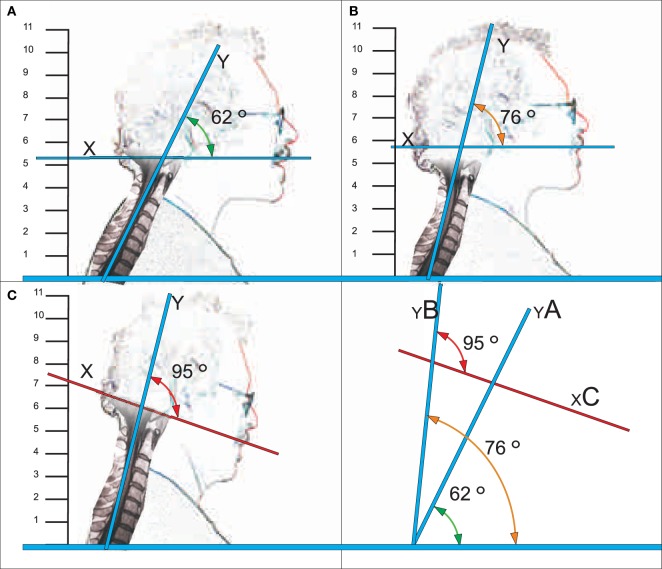
**Representation of tinnitus-related head postures and corresponding (posterior) craniocervical angles**. Position A: tinnitus neutral position, Position B: tinnitus trigger point, and Position C: tinnitus maximum strength.

After improvement of chronic tinnitus (see above and Figure 1), the tinnitus was *ad libitum* replicated with various constrained head postures but most easily with simple head inclination (spontaneously observed by the patient), and tinnitus severity was apparently increasing with stronger inclination. Therefore, tinnitus relevant head inclination was recorded with photographs, from these drawings made, then the inclination angles determined: graphically applying a tangent from the occipital extreme to the extreme of cervical lordosis (*Y*), and another line from the occipital extreme to the tip of the upper lips (*X*; Figure 2). Previous MR images, of the cervical spine were overlaid the illustrative drawings.

Tinnitus pitch was determined by a function generator (Heterodyne Analizer 2010, Brüel & Kjaer, Sound & Vibration Measurement A/S, Denmark) by comparing the generated tone to the subjective tinnitus pitch.

### Ultrasound Measurement of Blood Flow in Cerebral Arteries

Measurements of blood flow velocity in the major cerebral arteries were carried out by experienced neurologist (Gerhard F. Hanmann) [analogous method used as in Ref. (6, 7)] with the device EPIC 5, Phillips Company, and Multidop X (DWL Elektronische Systeme). Herewith, the changes in blood flow velocity can be indirectly related to changes in cerebral blood flow assuming that the diameter of the examined vessels did not change during the examination.

## Results

### Clinical Case Description

An acoustics technician (coauthor Martin Wieland) suffered for over 20 years from chronic tinnitus, starting with comorbid CPS and cervicogenic headache, initially, the CPS being in the foreground and at time even enhanced by unsuccessful osteopathic maneuvers (see Figure 1). Usual physiotherapies were then performed for longer time period and eventually the CPS improved, but not the tinnitus. All recommended therapies for tinnitus were for years without success, although, the patient visited several specialists who informed him to get used to tinnitus because it was untreatable. The non-pulsatile tinnitus (term as defined in 1) consisted of a continuous tone of 6–8 kHz frequency.

### Tinnitus Improvement by CC Trial

Despite the chronicity of about 20 years of tinnitus and the lack of a painful CPS in this case, Martin Wieland was informed about a speculative therapeutic chance, intermittent short time use of CC. Though hesitating, eventually the patient treated himself as an outpatient by CC as recommended (see Patients and Methods) with customized Basko CC, size L 3¼. Yet, 20 min after wearing CC for the first time, the patient felt a slight improvement of tinnitus; after wearing the CC over 2 weeks as recommended, usually about three times a day, for 15–30 min each, the tinnitus was significantly less distracting; this treatment schedule was continued for further 2 weeks, then the tinnitus remitted completely (see Figure 1). The patient did not need further treatment courses thereafter. Nevertheless, occasionally a slight resurgence of tinnitus was observed, namely, with longer constrained postures when associated with tension in the head–neck region, especially with head inclination alike with postures B and C (Figure 2). Improved subjective postural control and sometimes, partially renewed short-term application of CC (6 months after initiation of CC therapy about 1× for about 15 min every 2–4 weeks) led to rapid improvement of tinnitus after each CC application.

### Experimental Replication of Tinnitus-Related Head and Neck Angles

After clinical stable improvement, the patient observed that tinnitus might set on as often as he severely inclined his head. Under controlled conditions: tinnitus set on with anterior tilt of the head in position B and reached its maximum with position C (see Figure 2). With immediate return into initial position A, the tinnitus ended immediately. These inclination angles, measurable at the anterior or the posterior side, reflected the forward inclination of the head, apparently associated with increasing tension of the upper posterior craniocervical myofascial apparatus, from A to B to C.

### Cerebral Blood Flow Measurement by Ultrasound Methods

T duplex and TCD examination of vertebral arteries on both sides and the basilar artery in different head positions showed no blood flow changes in position B and C compared to A, see Figure 1, with neither the onset (B) nor the maximum of tinnitus experience (C). Nevertheless, turning of the head to the left or the right side, both postures, similarly provoked the expected physiological flow reduction in the respective vertebral artery of the contralateral side, respectively. However, these maneuvers did not trigger tinnitus.

## Discussion

We report on a case with chronic tinnitus of 20 years, which was considered to have arrived at the dead end of treatment in the judgment of several tinnitus specialists. We show here, that in this individual case, tinnitus was rapidly and completely remitted within 4 weeks with intermittent use of CC, despite the long-standing chronicity. Importantly, CC treatment was the exclusive treatment performed in the critical time period. This individual case observation, under other cases suffering from CPS accompanied by comorbid tinnitus, and previous own personal experiences (see Introduction and Patients and Methods), suggest an unknown therapeutic option of intermittent CC use in chronic tinnitus (maybe with CPS or without CPS). Also in case Martin Wieland reported here, the tinnitus was associated with painful CPS, at the time of tinnitus onset and during the first year of catamnesis. The CPS was then successfully treated, whereas the tinnitus remained therapy-resistant over the remaining 19 years. The time lag in this single case was considerable and the association tinnitus/CPS, therefore, may appear speculative. However, in many other cases, we observed tinnitus in association with painful CPS, similarly (unpublished). The rapid improvement of tinnitus with CC use in this single case may suggest a possible pathogenetic relationship of tinnitus to minor symptoms of cervical syndrome without pain, in that, yet, minor muscle tension of the upper posterior cranial transition may represent a sufficient tinnitus trigger. This assumption is supported in our case because after remission tinnitus could be spontaneously resumed with constrained head postures and was experimentally replicated, with increasing strength of tinnitus with increasing head inclination, the latter apparently associated with increasing tension within the UPCM. Overtly, individual UPCM tension levels for onset and for tinnitus maximum could be defined (head inclination angles). Interestingly, tinnitus set off immediately with relaxation. So, in principle, tinnitus seemed even *ad libitum* replicable with UPCM tension. Thus, the UPCM may well represent a special area to trigger tinnitus.

On the other hand, we could demonstrate that tinnitus was unrelated to cerebral blood flow in the vertebral arteries. Flow velocity was not altered during head inclination, whereas reduced during head rotation, as physiologically expected, but rotation (in contrast to inclination) was not associated with tinnitus. Therefore, blood brain perfusion was unrelated to tinnitus, whereas tension of the UPCM triggered tinnitus.

Our single case observation is rather preliminary with regard to technical methods but may nevertheless have some general relevance for hypothesis generation in tinnitus research, to be discussed in more details: from experiments in rats it was known that simple tension applied to the UPCM (as termed here analogously), elicited broad afferent reflex signal activity to the central nervous system, but only when applied to few upper posterior cervical muscles exclusively, whereas not when applied to other cervical muscles (8): the widespread alterations of CNS functioning exclusively induced by tension of the UPCM involved a newly described nucleus of the medulla responsible for autonomous functions, cardiovascular functions, and the vestibular system. In humans, the UPCM was not differentiated in analogous terminology, yet, though, the importance of the craniovertebral and cervicomedullar angles in the causation of cervicogenic headache was well described (9). The larger posterior craniovertebral angle with head inclination in our case (see Figure 2), respectively, the smaller anterior craniovertebral angle focused in the discussion by Coban et al (9), apparently associates with increased tension of the UPCM, which thus in principle may elicit both cervicogenic headache and/or cervicogenic tinnitus. Such interpretation would also match with established trigger points for cervicogenic headache (10) or tinnitus (11).

Bringing these findings together with our observations, we propose that cervicogenic tinnitus might be considered a special subtype of somatosensory tinnitus as described by others (3–5). The *ad libitum* experimental replication of tinnitus under controlled experimental conditions, together with demonstration of a dose effect, may even suggest a rather specific role of the UPCM, herein. The occasional recurrence of tinnitus in everyday life with constrained postures for longer time in our case (e.g., observed when working with personal computers, when driving on a mountain bike, etc.) elicited tension of the UPCM, though likely in concert with other muscles. Nevertheless, we consider the possibility that the UPCM may be coinvolved in various clinical situations described to trigger or to modulate tinnitus, like with gaze, voluntary muscle contractions in neck shoulder region, and trigger points in the neck region (12–15). Whether the UPCM might indeed represent a key trigger factor herein remains to be demonstrated, however.

In our single case, it remains to ask why chronic tinnitus could improve so quickly and completely with a simple exclusive therapeutic intervention with CC of 4 weeks. We suspect that a slight tightening of the UPCM was chronically prevalent though not producing clinically apparent CPS (nevertheless, at the very onset many years ago), but over years thereafter, just mild tension of the UPCM group may have prevailed. The only remaining symptom of the previous CPS recognized by the patient may, therefore, has been the chronic tinnitus, which was now rapidly relieved with CC treatment, by reducing the prevalent minor (not painful) UPCM tension. The application of a Basko CC, which is rather rigid and lifts the head up a little bit may then, due to the repeated transient weight off over a period of 4 weeks, have led progressively to a more lasting muscle relaxation of UPCM and thus, have ended a vicious circle. In other words, yet, minor chronic myofascial tension of the UPCM without pain may have been sufficient to trigger a low grade subtype of chronic somatosensory tinnitus. The occasional emergence of tinnitus after remission from CC treatment, then again, improved with occasional use of CC or posture control supports such interpretation. Such interpretation would also match with recent findings about CNS pathomechanisms in tinnitus (and hyperacusis), involving disinhibition and increased central gain in animal experiments (16), and in human cases (17). In both settings, widespread alterations activity within the CNS, not only within auditory networks, were involved. Thus, tension of the UPCM may, though in concert with other triggers including psychological/psychosomatic factors not discussed in detail here, play a prominent role in tinnitus pathomechanisms.

### Limitations

We could not perform exact measurements of the head–neck angles, measured only by drawings based on photographs. This simple method may have, nevertheless, been sufficient to grasp the principle. Most desired was to directly measure muscle tension and to define the muscles that were under tension, but such method is not available to our knowledge. We knew about the skepticism in neurotraumatology about the use of CC but short time intermittent use of CC was previously not tested and may be able to circumvent the problems of muscle inactivity and atrophy associated with continuous CC use.

In our case report, we were able to isolate, at least to a certain extent, one single factor, which allowed us to speculate about the possible weight of one, yet, rather unknown single factor, that is the potential role of UPCM tension. To isolate single factors is one important step in addressing the potential relevance of factors within an interactive network of factors. The next step, indeed, should be to place and discuss this single factor in the broad scenario of many “psychosomatic” factors. The latter had to be clearly defined as of their single specific weights and classified as more or less “psychic” or “somatic,” including a definition of the terms used. In addition, one had to differentiate that the relevance of single factors may be altered by interaction itself. Such undertaking requires an extended separate paper on the psychosomatics of tinnitus in the form of an extensive review, best written by several authors from various scientific fields involved.

In summary, intermittent short time use of CC might be a new treatment option of tinnitus even with chronicity. The pathogenetic mechanisms involved in tinnitus are not clear today. We suggest that tension of the UPCM may be an important trigger, also with a dose effect, and that tinnitus can be overcome by effective relaxation of the UPCM, because it was shown that UPCM tension is able to trigger reflex activity into the CNS and elicit widespread CNS activation, and such could match with known tinnitus pathomechanisms. Nevertheless, tinnitus may prevail in various clinical situations with muscle tension in the head–neck region, also influenced by psychological factors.

Experimental cervicogenic tinnitus was not associated with alterations of cerebral blood flow, and vice versa, the physiological reduction of vertebral artery blood flow with head turn was also not associated with tinnitus. This observation excludes the possibility that tinnitus was caused from altered cerebral blood flow, at least in our case.

Our single case observations may contribute to an emerging perspective about somatosensory tinnitus. We propose to perform systematic studies with better experimental equipment on a possible special role of the UPCM in tinnitus. The high overall prevalence of tinnitus within the population and the frequent prevalence of minor cervical syndromes (with or without pain), both prevailing apparently often in parallel, suggest a potentially broad relevance of our idea.

## Consent

All authors have read the final version of the paper and consented.

## Author Contributions

KB designed the performance of the study, created the idea, and wrote the article. MW described the patient, measured the aspects of tinnitus, created the figures, and was also involved in writing. GH measured the cerebral blood flow, participated in critical discussion, and contributed to writing of the article.

## Conflict of Interest Statement

None of the authors have conflicts of interest or potential conflicts of interests of any of the firms named in the paper.
